# Mechanistic Analysis of Decabromodiphenyl Ether-Induced Neurotoxicity in Humans Using Network Toxicology and Molecular Docking

**DOI:** 10.1007/s12640-025-00741-7

**Published:** 2025-03-24

**Authors:** Fuat Karakuş, Burak Kuzu

**Affiliations:** 1 Department of Pharmaceutical Toxicology, Faculty of Pharmacy, Van Yuzuncu Yil University, Van, Türkiye; 2 Department of Pharmaceutical Chemistry, Faculty of Pharmacy, Van Yuzuncu Yil University, Van, Türkiye; 3 Department of Pharmaceutical Toxicology, Faculty of Pharmacy, Van Yuzuncu Yil University, Tuşba-Van, 65080 Türkiye

**Keywords:** Decabromodiphenyl ether, Neurotoxicity, NMDA receptors, GluN2B

## Abstract

Commercial decabromodiphenyl ether (c-decaBDE) is a widely used additive flame retardant in textiles and plastics. This formulation predominantly consists of the congener BDE-209, with trace amounts of other brominated diphenyl ether congeners, such as nonabromodiphenyl ether and octabromodiphenyl ether. Recognized as a persistent organic pollutant due to its potential for long-range environmental transport, c-decaBDE poses significant environmental threats and serious human health risks, including endocrine, reproductive, developmental, and neurotoxic effects. The mechanisms underlying its neurotoxicity remain largely undefined. This study investigates the neurotoxic effects of BDE-209 in humans through network toxicology, multi-level bioinformatics approaches, and molecular docking analyses. Prediction results indicate that BDE-209 can cross the blood-brain barrier, entering the central nervous system and inducing neurotoxic effects. A comprehensive analysis has identified 294 potential targets linked to the neurotoxicity induced by BDE-209. Gene-gene interaction and pathway enrichment analyses revealed significant associations related to cellular responses to chemical stress and synaptic transmission. Further investigation of protein-protein interactions, combined with centrality analysis, identified 14 hub targets, including CaMK-II alpha, PSD-95, GluR-1, and GluN2B, as key proteins in this process. Molecular docking results indicate that BDE-209 exhibits a stronger binding affinity to GluN2B, a subunit of the N-methyl-D-aspartate (NMDA) receptors, compared to other key targets. These findings suggest that BDE-209 may disrupt the function of GluN2B-containing NMDA receptors, potentially leading to their inhibition. Such inhibition could result in reduced excitatory neurotransmission, impairing synaptic potentiation and plasticity, and ultimately contributing to neurotoxicity.

**Supplementary Information:**

The online version contains supplementary material available at 10.1007/s12640-025-00741-7.

## Introduction

The commercial mixture decabromodiphenyl ether (c-decaBDE) is widely utilized as an additive flame retardant in textiles and plastics, with additional applications in adhesives as well as coatings and inks. C-decaBDE is primarily composed of the congener BDE-209 (≥ 97%), with trace amounts of other brominated diphenyl ether congeners, such as nonabromodiphenyl ether (0.3-3%) and octabromodiphenyl ether (0-0.04%) (UNEP, 2014).

The long-range environmental transport of BDE-209 and its debromination to lower brominated products have significant adverse effects on wildlife, including neurobehavioral alterations in zebrafish offspring (He et al. 2011), developmental issues such as delayed metamorphosis in amphibians, and disruptions in thyroid function in fish (UNEP, 2014). Furthermore, developmental neurotoxicity has been observed in terrestrial mammals (Rice et al. 2007; Reverte et al. 2013; Xu et al. 2018; Eriksson et al. 2023). In terms of human health, BDE-209 has been linked to various risks, including endocrine disruption, developmental and reproductive health issues, and neurotoxicity (Kiciński et al. 2012; UNEP, 2014; Martin et al. 2017; Vuong et al. 2018; Wang et al. 2024). Research has shown that BDE-209 is absorbed and distributed in human fat, blood, cord blood, placenta, fetuses, and breast milk. Major sources of human exposure to BDE-209 include dust, indoor air, infant formula, and food (UNEP, 2014; EFSA, 2024; Milić et al. 2024). Consequently, BDE-209 was proposed for inclusion in the Stockholm Convention on Persistent Organic Pollutants and was officially listed in Annex A of the Convention SC-8/10 in 2017, with specific exemptions (UNEP, 2017).

Both in vitro and in vivo studies and epidemiological observations in humans suggest that BDE-209 may be neurotoxic to humans (Chao et al. 2011; Gascon et al. 2012; Linares et al. 2015; Mariani et al. 2015; Chevrier et al. 2016; Xu et al. 2018; Vuong et al. 2018; Eriksson et al. 2023; Sun et al. 2024). Given these findings, it is essential to understand the mechanisms underlying the neurotoxic effects of BDE-209 on humans in order to accurately assess its health risks and establish appropriate safety guidelines.

Computational toxicology has emerged as a crucial tool for elucidating neurotoxicity and other toxicities induced by xenobiotics. It allows for the exploration of complex interactions between chemical compounds and biological systems. This field utilizes computational models to integrate various omics data, offering a comprehensive understanding of how toxicants disrupt cellular networks and lead to injuries. Furthermore, bioinformatics techniques, such as pathway analysis, aid in identifying key targets and mechanisms linked to toxicity, thereby providing insights into the molecular processes underlying adverse effects. These methodologies present a holistic view of chemical toxicity, facilitating the discovery of biomarkers and potential therapeutic interventions (Nguyen and Kim 2023; Karakuş et al. 2024).

This study specifically examines the role of BDE-209 in inducing neurotoxicity and investigates the mechanisms underlying its neurotoxic effects in humans. Utilizing network toxicology, bioinformatics, and molecular docking techniques, the research integrates data on protein-protein interactions and metabolic pathways to elucidate the molecular mechanisms behind BDE-209’s neurotoxic effects. The study also aims to identify key targets associated with neurotoxicity induced by BDE-209.

## Materials and Methods

### Investigated Compound

In this research, BDE-209 (CAS number: 1163-19-5) was examined, and its SMILES codes were obtained from the PubChem database (Kim et al. 2023). BDE-209 underwent toxicity assessments using ADMETlab 3.0 (Fu et al. 2024), which utilized SMILES to evaluate their permeability through the blood-brain barrier (BBB) and potential human neurotoxicity. ADMETlab 3.0 incorporates a deep learning approach that utilizes a graph neural network based on a Directed Message Passing Neural Network framework for predictions. BBB classification was based on logBBB values, with compounds exhibiting logBBB > -1 categorized as BBB-permeable (BBB+, Category 1), and those with logBBB ≤ -1 designated as non-permeable (BBB-, Category 0). The probability score, ranging from 0 to 1, reflects the likelihood of a compound being BBB+. In the context of drug-induced neurotoxicity, predicted neurotoxicity values fall within a spectrum from non-toxic (0.3 and below) to toxic (0.7–1.0). However, the permeability of the BBB alone does not determine toxicity, as numerous compounds can also be substrates for P-glycoprotein-mediated efflux from the brain. Consequently, central nervous system (CNS) permeability was also estimated using Deep-PK (Myung et al. 2024).

### Curation of BDE-209 Targets

The potential targets of BDE-209 related to *Homo sapiens* were sourced from the Comparative Toxicogenomics Database (CTD) (Davis et al. 2023), the PubChem database (Kim et al. 2023), and the SwissTargetPrediction tool (Daina et al. 2019). The structural information obtained from all sources was carefully cross-validated for consistency. Subsequently, all targets from these databases were merged, deduplicated, and their gene names were standardized using the UniProt database (UniProt Consortium 2023), resulting in the creation of a BDE-209 target library.

### Construction of the Neurotoxicity-Associated Targets Network

Extensive searches were conducted across the CTD (Davis et al. 2023), DisGeNET (Piñero et al. 2020), GeneCards (Stelzer et al. 2016), and OMIM (Amberger et al. 2019) databases to identify relevant targets associated with neurotoxicity. The keyword “neurotoxicity” was used for this purpose, resulting in a curated library of unique targets. Additionally, transcriptional profile datasets from the NCBI GEO database (Clough et al. 2024) were selected for further analysis, specifically GSE33109, GSE199502, and GSE70845. The thresholds for determining differentially expressed genes (DEGs) were set at|Log2 (FC)| > 1 and an adjusted p-value < 0.05.

Subsequently, a Venn diagram was created using the Jvenn tool (Bardou et al. 2014) to illustrate the overlap between the predicted neurotoxicity targets and the DEGs from the neurotoxicity datasets. This subset of overlapping targets was considered a potential contributor to human neurotoxicity.

Finally, another Venn diagram was generated to depict the intersecting targets between the potential neurotoxicity targets and those related to BDE-209. This subset of overlapping targets was considered potential candidates for BDE-209-induced neurotoxicity in humans.

### Gene-Gene Interactions and Pathway Analysis of Potential Targets

The interactions between genes for potential targets were examined through GeneMANIA, which uses a vast dataset of functional relationships to pinpoint genes related to a given set of input genes. These relationships encompass protein and genetic interactions, pathways, co-expression, co-localization, and similarities in protein domains (Warde-Farley et al. 2010). The analysis centered on the target organism *Homo sapiens*, and the network weighting was carried out using the automatically chosen weighting method.

The pathways associated with potential targets in BDE-209-induced neurotoxicity was elucidated through REACTOME pathway enrichment analyses. The analysis was conducted using the DAVID database (Sherman et al. 2022), which provides significant REACTOME pathways related to the potential targets of BDE-209-induced neurotoxicity. Results with a p value < 0.05 were considered statistically significant. The top 10 terms from REACTOME analyses were visualized using SRplot (Tang et al. 2023).

### Construction of Protein-Protein Interaction (PPI) Networks and Identification of Key Targets

To evaluate the potential targets of neurotoxicity induced by BDE-209, the STRING database (Szklarczyk et al. 2015) was employed to outline the corresponding proteins and their interaction networks. The parameters for analysis were rigorously established to restrict the organism to *Homo sapiens*, necessitating a minimum interaction score exceeding 0.9 (which indicates the highest level of confidence). This approach ensured a concentrated analysis of the active proteins linked to the target genes. Data from STRING were later imported into Cytoscape software (version 3.10.3) (Shannon et al. 2003) to calculate the topological properties of network nodes and edges, visualize molecular connections, and construct the PPI network diagram. Four different algorithms—maximal clique centrality (MCC), maximum neighborhood component (MNC), Degree centrality metrics through the cytoHubba plugin, and the Molecular Complex Detection (MCODE) method from Metascape (Zhou et al. 2019)—were utilized to assess node significance scores and identify critical hub targets. Targets recognized by at least two of these algorithms were classified as hub targets. By merging hub targets with a second MCODE analysis in Metascape, the primary hub targets were determined as key targets.

### Molecular Docking

Molecular docking analyses were performed using AutoDock 4.2 (Morris et al. 2009) to assess the binding affinity and interaction patterns between BDE-209 and its target proteins. The molecular structure of BDE-209 was obtained from PubChem (Kim et al. 2023), and the 3D structures of experimentally determined proteins were sourced from the RCSB Protein Data Bank (www.rcsb.org). For proteins without PDB structures, 3D models were retrieved from AlphaFold DB (Varadi et al. 2024). The crystal co-ligands with BDE-209 were modeled using Gaussview 5.0 and optimized via DFT with Gaussian 03 (B3LYP method, 6–31G basis set). Docking simulations used PDB IDs 2VZ6 (Rellos et al. 2010) and 5EWJ (Stroebel et al. 2016), and AlphaFold DB IDs AF-P78352-F1-v4 and AF-P42261-F1-v4 for missing structures.

Solvents, water, and ligands were removed, and polar hydrogen atoms and Gasteiger and Kollman charges were added. The grid box was positioned to cover the target regions, allowing free molecular movement. Docking centers were defined as: 2VZ6 (x: 51.85, y: 60.689, z: 18.468), and 5EWJ (x: 23.07, y: -4.402, z: -32.688). The docking pocket was defined as a cubic 40 Å × 40 Å × 40 Å, with 0.375 Å grid spacing. For AlphaFold proteins, blind docking was used. The Lamarckian genetic algorithm was applied with 10 runs and a population size of 300. The docking procedure was validated by re-docking the native ligand and comparing it with the crystal structure. The conformation with the highest receptor affinity was selected, and binding energy was calculated. Interactions between the protein structures and BDE-209 were analyzed in 2D and 3D formats using Discovery Studio Client 4.1.

## Results

### Predictive Neurotoxicity of BDE-209

Computational models predict that BDE-209 may cross the BBB, infiltrate the CNS, and potentially cause neurotoxicity (Table 1). The model-estimated neurotoxicity probability is 0.94. The compound is predicted to have a high probability of crossing the BBB, as indicated by the +++ BBB penetration classification (corresponding to a predicted BBB + probability range of 0.9–1.0). Additionally, the calculated logPS value for BDE-209 (-0.38) suggest potential permeability to the CNS according to Deep-PK results. These in silico predictions indicate that, BDE-209 may exhibit neurotoxic potential.

**Table 1:** Predicted neurotoxicity potential of BDE-209

Compounds	CAS Number	Canonical SMILES	ADMETlab 3.0 (https://admetlab3.scbdd.com)		Deep-PK (https://biosig.lab.uq.edu.au/deeppk/)
			Drug-induced Neurotoxicity	BBB penetration	Central nervous system (logPS)
BDE-209	1163-19-5	C1(= C(C(= C(C(= C1Br)Br)Br)Br)Br)OC2 = C(C(= C(C(= C2Br)Br)Br)Br)Br	0.94	+++	-0.38

**Neurotoxicity**: Non-toxic (0–0.3), Moderate (0.3–0.7), Toxic (0.7–1.0). **BBB penetration**: +++ reflects BBB + probability (0.9–1.0). **Deep-PK CNS permeability**: logPS indicates permeability-surface area; CNSp+ (logPS ≥ − 2) and CNSp− (logPS ≤ − 3) denote classification

### Acquisition of Targets in BDE-209-Induced Neurotoxicity

**Fig. 1:**
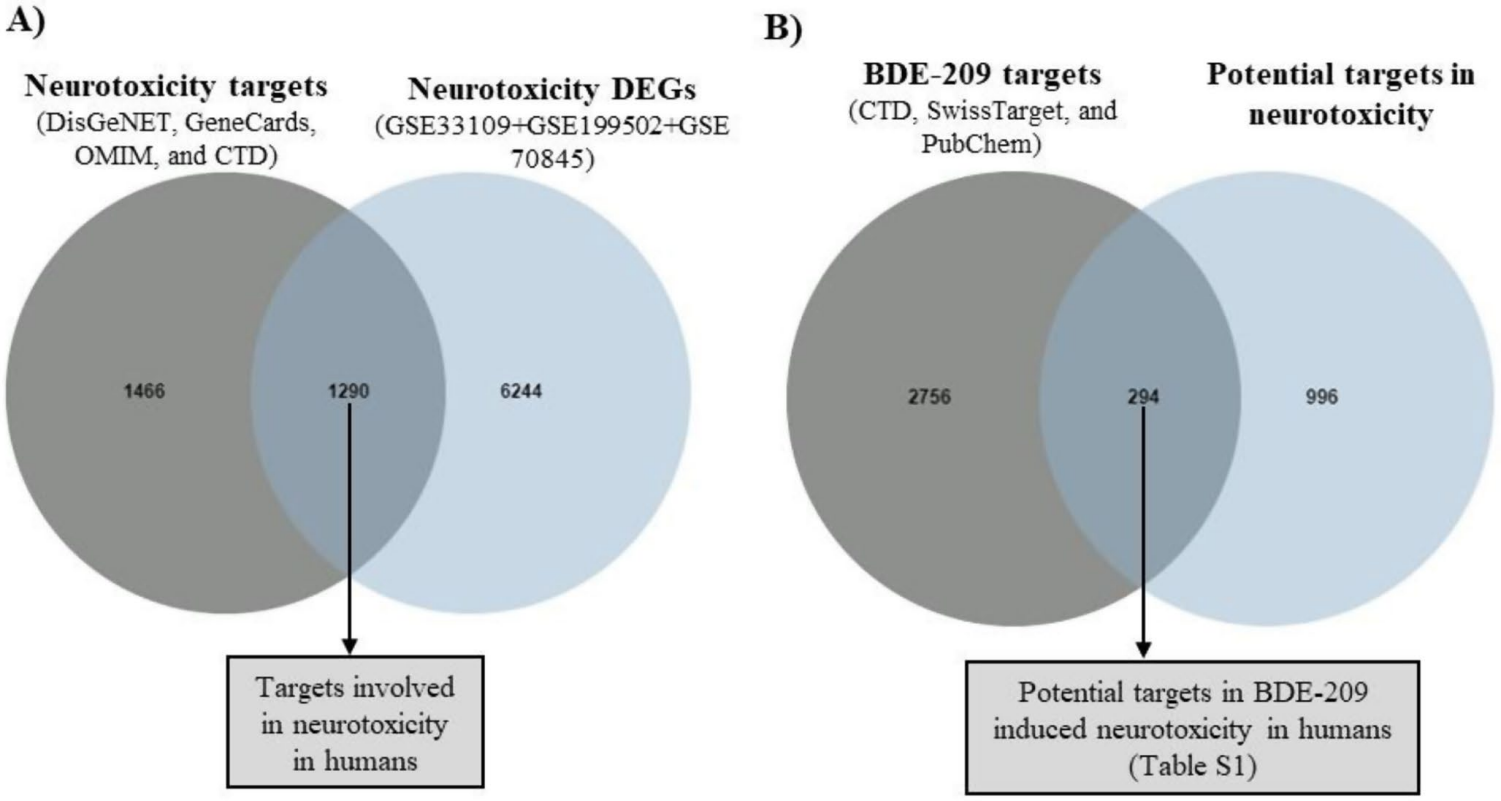
Venn diagram of targets for BDE-209-induced neurotoxicity in humans. **(A)** The brown circle represents 2756 neurotoxicity targets, while the blue circle represents 7534 DEGs associated with neurotoxicity in humans. The overlapping region reveals 1290 targets involved in neurotoxicity in humans **(B)** The brown circle represents 3050 BDE-209 targets, while the blue circle represents 1290 neurotoxicity targets in humans. The overlapping region reveals 294 targets associated with both BDE-209 and neurotoxicity, indicating potential targets for BDE-209-induced neurotoxicity in humans

In this study, a total of 2756 neurotoxicity targets were initially curated by integrating data from DisGeNET, GeneCards, OMIM, and CTD. Concurrently, 7534 DEGs were identified from neurotoxicity-related datasets referenced as GSE33109, GSE199502, and GSE70845. Utilizing an integrative Venn diagram approach, 1290 overlapping targets were identified as potentially relevant to neurotoxicity in humans (Fig. 1A). Furthermore, 3050 targets associated with BDE-209 were uncovered from the CTD, SwissTarget, and PubChem databases. By applying another Venn diagram, 294 overlapping targets were identified and regarded as potential targets in BDE-209-induced neurotoxicity in humans (Fig. 1B). These potential targets are listed alphabetically in Table S1.

### Gene-Gene Interaction Network and Pathway Enrichment of Potential Targets

**Fig. 2:**
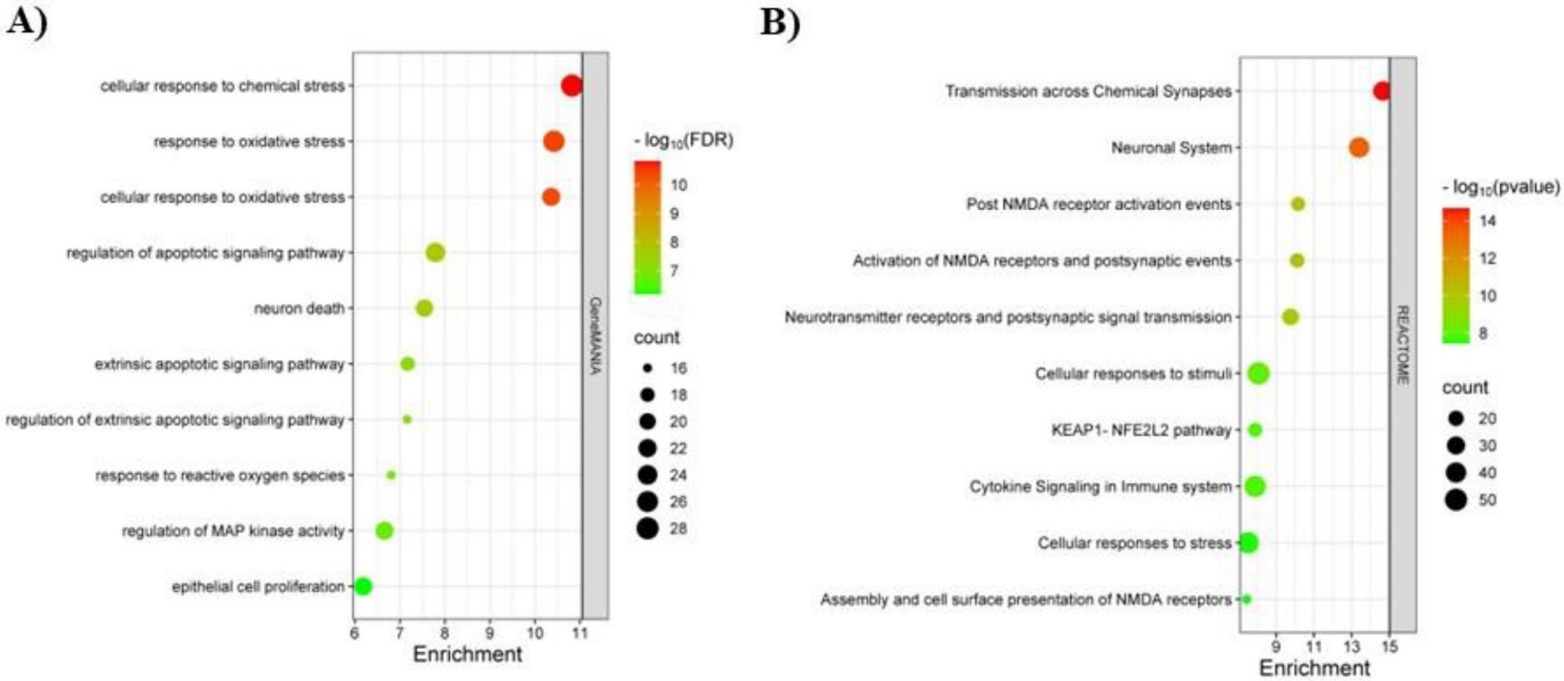
Gene-gene interactions and pathway enrichment analysis of 294 potential targets. Each bubble represents a function or pathway, with its size proportional to the number of enriched targets. The color intensity indicates the significance of enrichment **(A)** The bubble plot displays the top 10 functions associated with 294 potential genes. **(B)** The bubble plot displays the top 10 most enriched REACTOME pathways associated with 294 potential targets

Following the identification of 294 potential targets (Fig. 1B), their gene-gene interactions were analyzed using GeneMANIA. The official gene symbols were input into the GeneMANIA online plugin to construct a comprehensive network. The functional enrichments analysis of these 294 targets revealed a primary association with cellular responses to chemical stress (Fig. 2A, Table S2). The interaction network analysis indicated that co-expression (42.43%) and physical interactions (38.69%) were the predominant factors among these 294 genes (Fig. S1). Co-expression suggests that these genes are likely to be expressed together, indicating their involvement in similar biological processes or regulatory pathways. Physical interactions imply that the proteins encoded by these genes physically associate, potentially forming complexes or collaborating in cellular functions.

To further investigate, REACTOME pathway analysis was conducted on the 294 potential targets using the DAVID database, uncovering 269 enriched pathways associated with these targets. A bubble chart, organized by *p*-value to illustrate the ten most enriched REACTOME pathways and arranged by the percentage of gene counts, was generated (Fig. 2B). Notably, a significant association was identified among pathways related to “Transmission across Chemical Synapses,” the “Neuronal System,” and N-methyl D-aspartate (NMDA) receptors, suggest that the identified targets may be involved in neurobiological processes relevant to chemical exposure, including BDE-209. Detailed pathway terms are presented in Table S3.

### PPI Network of Potential Targets and Key Target Identification

**Fig. 3:**
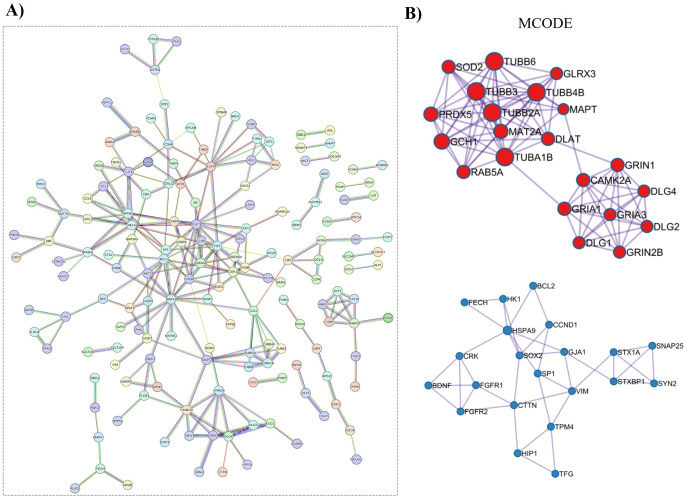
PPI network of 294 potential targets and MCODE analysis. **(A)** The PPI network from STRING with a highest confidence score (0.9). **(B)** Hub and key target candidates identified through MCODE algorithms via Metascape

The 294 potential targets linked to BDE-209-induced neurotoxicity were mapped onto a PPI network using the STRING database with a confidence score of 0.9 (Fig. 3A). This analysis resulted in an interconnected network consisting of 293 nodes and 248 edges, yielding an average node degree of 1.69 and a PPI enrichment *p*-value of less than 1.0 × 10^-16^. Subsequently, the MCODE algorithm from Metascape was employed on the STRING PPI network, revealing six clusters, two of which were associated with neurotoxicity (Fig. 3B).

**Fig. 4:**
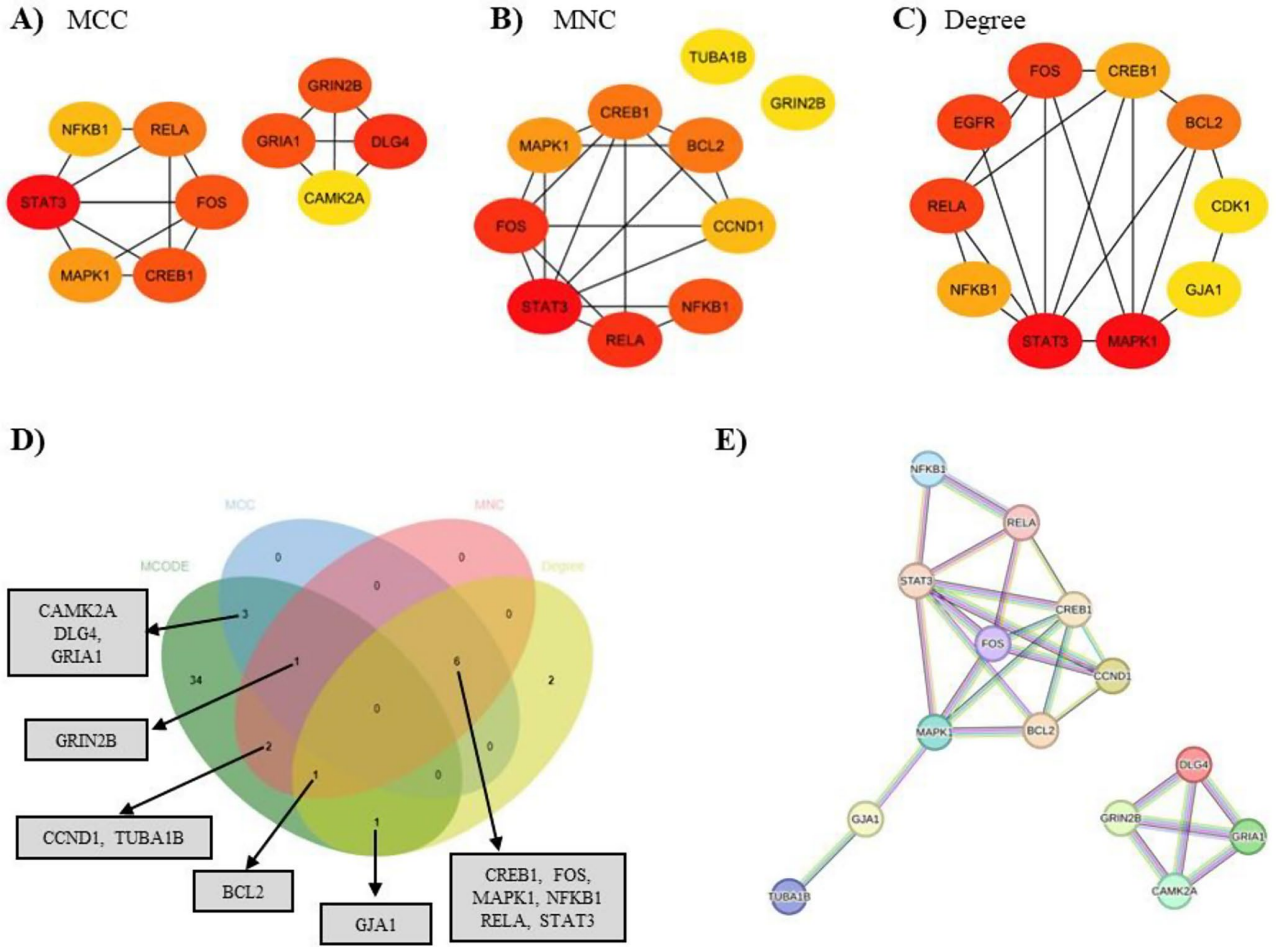
Hub and key targets identification via cytoHubba plugin of Cytoscape. **(A-C)** Hub and key target candidates identified through MCC, MNC, Degree. The color intensity of the nodes was correlated with their calculated degree, with more intensely colored nodes representing higher degree values. **(D)** Hub targets identified by at least two of the MCODE, MCC, MNC, and Degree algorithms. **(E)** PPI network of 14 hub targets

Additionally, a range of complementary algorithms, including MCC (Fig. 4A), MNC (Fig. 4B), and Degree (Fig. 4C) from the cytoHubba plugin of Cytoscape, were utilized to identify highly connected hub targets within the PPI network. Targets identified as hubs by at least two of these four methods (MCODE, MCC, MNC, and Degree) were selected, resulting in a subset of 14 targets closely related to BDE-209-induced neurotoxicity (Fig. 4D). A corresponding PPI network for these 14 targets was also constructed (Fig. 4E).

These 14 hub targets underwent a comprehensive pathway enrichment analysis using the DAVID database, revealing 163 significantly enriched pathways, with a particular emphasis on NMDA receptors. The top ten most enriched pathways are presented alongside their *p*-values in Table 2.

**Table 2:** Pathway enrichment analysis of 14 hub targets

REACTOME term	*p* value	Genes
Post NMDA receptor activation events	1.90 × 10^-10^	CREB1/CAMK2A/DLG4/GRIA1/GRIN2B/MAPK1/TUBA1B
Activation of NMDA receptors and postsynaptic events	4.70 × 10^-10^	CREB1/CAMK2A/DLG4/GRIA1/GRIN2B/MAPK1/TUBA1B
Estrogen-dependent nuclear events downstream of ESR-membrane signaling	1.20 × 10^-08^	BCL2/CREB1/CCND1/FOS/MAPK1
Cytokine Signaling in Immune system	2.00 × 10^-08^	BCL2/CAMK2A/CCND1/CREB1/FOS/MAPK1/NFKB1/RELA/STAT3/TUBA1B
CREB1 phosphorylation through NMDA receptor-mediated activation of RAS signaling	2.60 × 10^-08^	CAMK2A/CREB1/DLG4/GRIN2B/MAPK1
Neurotransmitter receptors and postsynaptic signal transmission	5.50 × 10^-08^	CREB1/CAMK2A/DLG4/GRIA1/GRIN2B/MAPK1/TUBA1B
Signal Transduction	2.50 × 10^-07^	BCL2/CREB1/CAMK2A/CCND1/DLG4/FOS/GJA1/GRIN2B/MAPK1/NFKB1/RELA/STAT3/TUBA1B
Signaling by Interleukins	2.70 × 10^-07^	BCL2/CREB1/CCND1/FOS/MAPK1/NFKB1/RELA/STAT3
Transmission across Chemical Synapses	2.80 × 10^-07^	CREB1/CAMK2A/DLG4/GRIA1/GRIN2B/MAPK1/TUBA1B
Cellular responses to stress	6.10 × 10^-07^	BCL2/CREB1/CAMK2A/FOS/MAPK1/NFKB1/RELA/STAT3/TUBA1B

A second MCODE analysis via Metascape applied to the PPI network linked to these 14 hub targets (Fig. 4E) identified four key targets, presented in Table 3.

**Table 3:** Four key targets related to BDE-209-induced neurotoxicity

Name	Protein Symbol	Gene Symbol
Calcium/calmodulin-dependent protein kinase type II subunit alpha	CaMK-II alpha	CAMK2A
Disks large homolog 4	PSD-95	DLG4
Glutamate receptor 1	GluR-1	GRIA1
Glutamate receptor ionotropic, NMDA 2B	GluN2B (NR2B)	GRIN2B

**Fig. 5:**
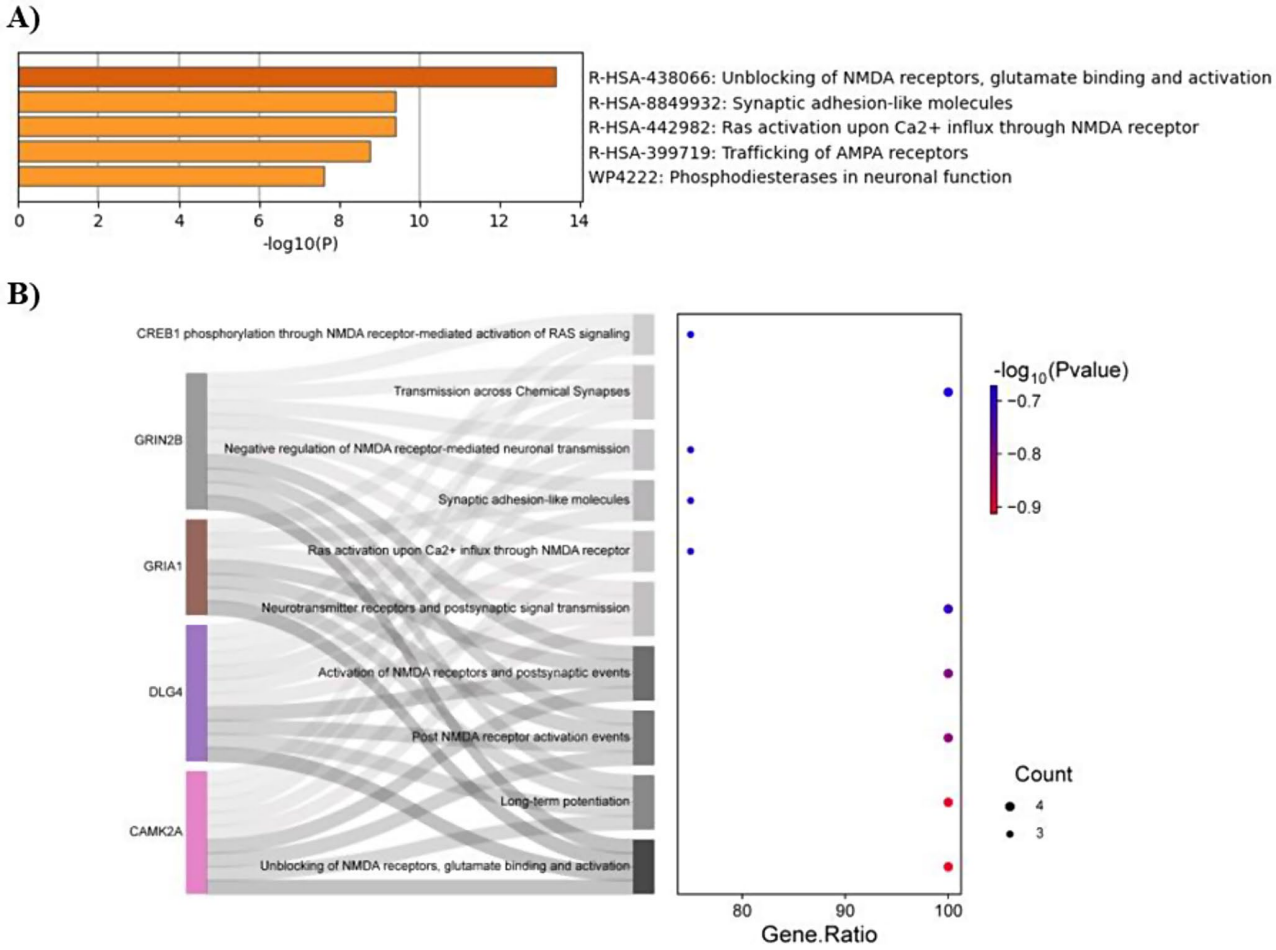
Pathway enrichment analysis for four key targets. **(A)** The histogram illustrates the most associated pathways identified by Metascape for the four key targets **(B)** The Sankey diagram and dot plot present the top 10 most enriched REACTOME signaling pathways, ranked according to their *p*-values from the DAVID database. Each dot represents a specific pathway and is connected by grey lines. The size of each dot indicates the number of enriched genes associated with that pathway

These four key targets were found to be associated with the unblocking of NMDA receptors, as well as glutamate binding and activation (Fig. 5A). Pathways related to these four targets were sourced from both Metascape and the DAVID database, and a Sankey-dot blot visualizing the top ten enriched REACTOME terms ranked by *p*-values was created using SRplot (Fig. 5B). Detailed pathway terms are presented in Table S4.

### Molecular Docking of BDE-209 against Key Targets

A molecular docking analysis was performed to evaluate the binding affinity of BDE-209 with key targets, including CaMK-II alpha, PSD-95, GluR-1, and GluN2B. The binding poses and interactions of BDE-209 with these targets were assessed using AutoDock 4.2 tools, resulting in the determination of binding energy values for each interaction. All binding energies for the targets were ≤ -7 kcal/mol, suggesting a relatively stable interaction under the docking conditions. Furthermore, a Root Mean Square Deviation (RMSD) of less than 2.0 Å is generally considered acceptable, while an RMSD of less than 1.0 Å is regarded as excellent, reflecting very high accuracy (Table 4).

**Table 4:** Binding energies of BDE-209 and control compounds against target proteins

Target	PDB ID	Compound	Binding energy (kcal/mol)	RMSD (Å)
CaMK-II alpha	2VZ6	BDE-209	-7.00	1.29
		FEF (Co-ligand)	-9.04	1.36
PSD-95	AF-P78352-F1-v4	BDE-209	-7.39	0.80
GluR-1	AF-P42261-F1-v4	BDE-209	-7.55	0.47
GluN2B	5EWJ	BDE-209	-8.99	0.42
		Ifenprodil (Co-ligand)	-9.93	0.59

**Fig. 6:**
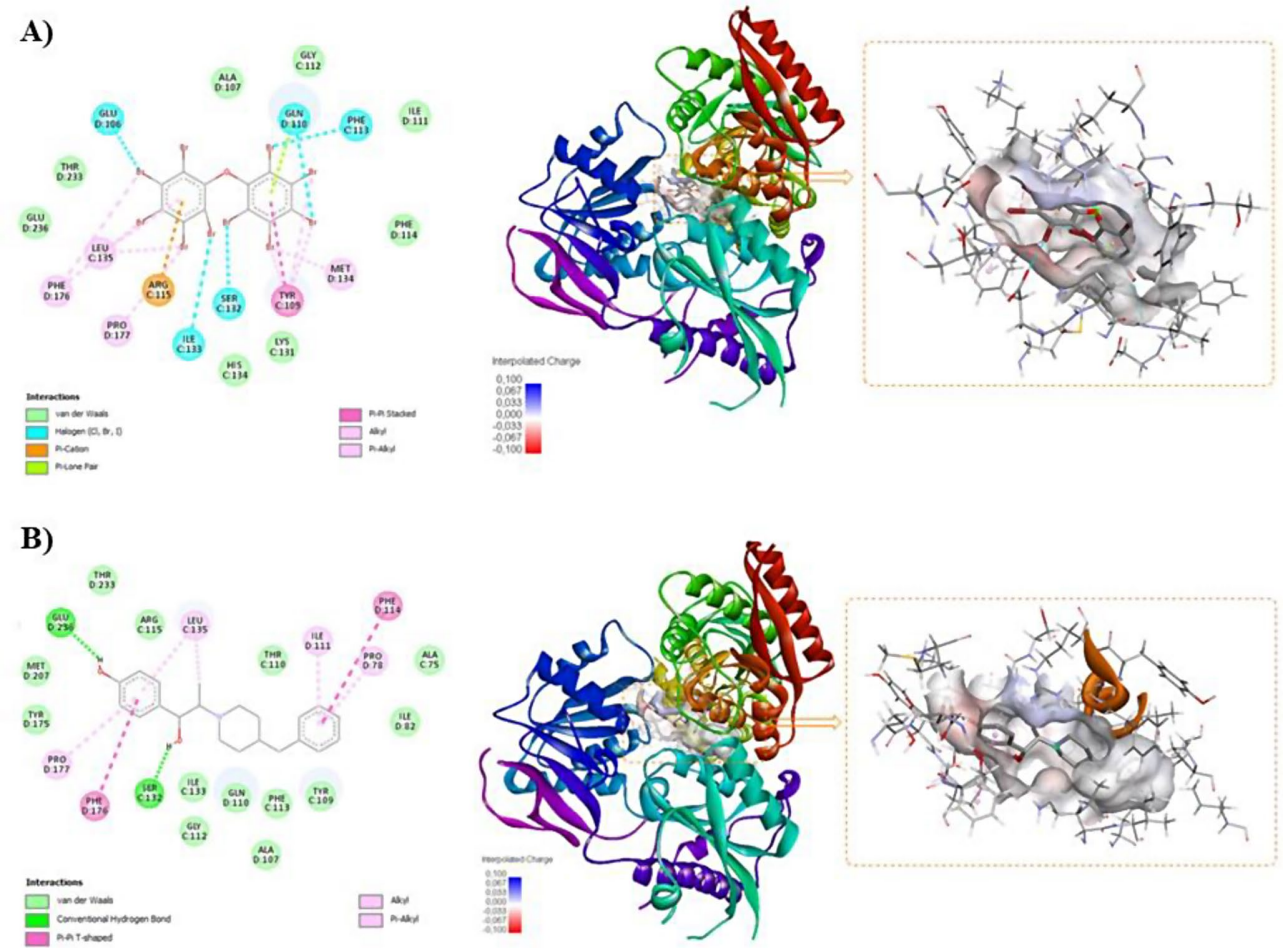
Molecular docking results of GluN2B (PDB ID: 5EWJ). **(A)** 2D and 3D interactions between BDE-209 and GluN2B **(B)** 2D and 3D interactions between Ifenprodil and GluN2B

The results demonstrate that BDE-209 has a moderate binding affinity for PSD-95 and GluR-1, while showing a relatively higher binding affinity for GluN2B (− 8.99 kcal/mol) with a highly reliable pose (RMSD = 0.42 Å). Ifenprodil, a selective inhibitor of the NMDA receptor, particularly for GluN2B subunit, showed a binding affinity of − 9.93 kcal/mol, which is slightly lower in energy compared to BDE-209 (Table 4). The 2D and 3D interactions of BDE-209 and Ifenprodil with GluN2B are presented in Fig. 6A and B, respectively.

## Discussion

The study presents evidence linking the persistent organic pollutant BDE-209 to neurotoxicity in humans. Based on computational modeling, BDE-209 demonstrated a high probability of crossing the BBB, penetrating the CNS, and potentially induce neurotoxicity in humans (Table 1). Some in vivo studies on BBB penetration have reported that small amounts of BDE-209 residues can cross the blood-brain barrier (Riu et al. 2008) and have been detected in the brain (Zheng et al. 2015). Regarding neurotoxicity, in silico results (Table 1) are supported by numerous in vitro and in vivo studies, as well as epidemiological observations (UNEP, 2014; Chao et al. 2011; Gascon et al. 2012; Linares et al. 2015; Mariani et al. 2015; Chevrier et al. 2016; Xu et al. 2018; Vuong et al. 2018; Eriksson et al. 2023; EFSA, 2024).

By employing an integrative approach that combines network toxicology and bioinformatics revealed 1290 targets play role in neurotoxicity in humans (Fig. 1A) and 294 potential targets were common between these neurotoxicity targets and BDE-209 associated targets (Fig. 1B, Table S1). Gene-gene interactions among 294 potential targets revealed a significant association with cellular responses to chemical stress (Fig. 2A, Table S2) with mostly co-expressed (42.43%) and physically interacted (38.69%) (Fig. S1). These means that these 294 genes may function together in related biological processes or regulatory pathways, and might associate to form complexes or cooperate in cellular activities. A recent in vivo study reported that BDE-209 elevated reactive oxygen species and malondialdehyde levels while reducing glutathione levels in the hippocampus of mice at both low and high doses (Wang and Dai 2022). Additionally, some in vitro studies suggest that BDE-209 decreases cell proliferation through oxidative stress in neurons (Chen et al. 2010; Yang et al. 2021).

Furthermore, REACTOME pathway enrichment analyses suggest a potential link between BDE-209-induced neurotoxicity in humans and the “Transmission across Chemical Synapses,” the “Neuronal System,” and NMDA receptors (Fig. 2B, Table S3). NMDA receptors are ligand-gated cation channels that mediate the voltage-dependent opening of a transmembrane cation pore in response to glycine and glutamate binding. The resulting calcium influx is essential for various processes, including neuronal development, synaptic plasticity, learning, and memory consolidation. These receptors operate as obligate heterotetramers, typically composed of two GluN1 subunits alongside either two GluN2 subunits (which may be of identical or differing subtype—GluN2A-D) or a combination of one GluN2 and one GluN3 subunit with two GluN1 subunits. Dysregulation of NMDA receptor function has been implicated in a range of neuropsychiatric and neurodegenerative conditions, such as Alzheimer’s disease, depression, schizophrenia, and stroke-induced ischemic injuries (Regan et al. 2015; Hansen et al. 2021).

Through PPI and centrality analysis of 294 potential targets, 14 hub proteins predominantly associated with NMDA receptors were identified (Table 2). Among these, CaMK-II alpha, PSD-95, GluR-1, and GluN2B emerge as key targets implicated in BDE-209-induced neurotoxicity in humans (Figs. 3 and 4). Pathway enrichment analysis suggested that these four critical proteins (Table 3) are primarily involved in the “Unblocking of NMDA receptors, glutamate binding, and activation” (Fig. 5, Table S4). Under resting membrane potential, NMDA receptors are obstructed by extracellular Mg²⁺ ions, preventing ion permeation. This voltage block is lifted upon depolarization of the post-synaptic cell membrane, leading to the expulsion of Mg²⁺ from the NMDA receptors and resulting in the activation of ligand-bound NMDA receptors (Hansen et al. 2021). BDE-209 may contribute to neurotoxicity in humans by disrupting the function of four key proteins—CaMK-II alpha, PSD-95, GluR-1, and GluN2B—for NMDA receptor activation. A recent in vivo study demonstrated that developmental exposure to BDE-209 disrupted NMDAR-dependent glutamatergic excitatory signaling, primarily by inhibiting GluN2B subunit activity. This impairment in receptor function contributed to deficits in learning and memory, highlighting NMDA receptor perturbation as a key mechanism underlying BDE-209-induced neurotoxicity (Shen et al. 2025).

Molecular docking analyses indicate that BDE-209 has moderate binding affinity for PSD-95 and GluR-1, with relatively higher binding affinity for GluN2B, as shown by PDB ID: 5EWJ (Table 4; Fig. 6). The GluN2B protein, which is encoded by the *GRIN2B* gene, serves as a subunit of NMDA receptors and plays a crucial role in synaptic plasticity, learning, and memory. During early development, GluN2B-containing NMDA receptors are more prevalent and are gradually supplanted by GluN2A in mature neurons (Gambrill and Barria 2011). BDE-209’s inhibition of GluN2B was comparable to that of Ifenprodil, a selective GluN2B inhibitor (Table 4), suggesting that BDE-209 may exert an inhibitory effect on NMDA receptors. Additionally, Xiong et al. (2018) reported that exposure to BDE-209 led to a decrease in GluN2B protein expression in rats, which was associated with spatial learning and memory impairment. Reduced NMDA receptor activity may hinder excitatory neurotransmission and disrupt synaptic potentiation and plasticity, potentially impairing long-term potentiation, a process essential for memory formation (Xiong et al. 2018).

Overall, these findings suggest that GluN2B inhibition caused by BDE-209 plays a critical role in BDE-209-induced neurotoxicity in humans by reducing the activation of GluN2B-containing NMDA receptors.

This study utilizes advanced computational tools in conjunction with experimentally derived NCBI GEO datasets to predict BBB permeability, neurotoxicity, target identification, and molecular interactions. However, the in silico methods employed present several limitations. The deep learning frameworks that support ADMETlab 3.0 and Deep-PK may inadequately predict the properties of novel chemical entities. Furthermore, BBB permeability predictions based solely on logBBB values do not take into account active transport mechanisms, particularly P-glycoprotein efflux. Target prediction resources such as the CTD, GeneCards, DisGeNET, and OMIM face accuracy limitations due to incomplete datasets and the possibility of false positive identifications. Therefore, future studies should incorporate experimental validation, particularly for BDE-209 and GluN2B, to confirm computational predictions and address these limitations.

## Conclusion

This study employed in silico tools to explore the neurotoxic potential of BDE-209 and its underlying mechanisms in humans. The results demonstrated that BDE-209 possesses neurotoxic properties. Further computational analyses and comprehensive bioinformatics approaches identified 294 potential targets associated with BDE-209-induced neurotoxicity, with four key proteins standing out as particularly significant. Docking studies confirmed that BDE-209 binds to these targets, especially highlighting GluN2B, which is believed to play a crucial role in mediating the neurotoxic effects of BDE-209. Overall, the findings suggest that exposure to BDE-209 may primarily lead to neurotoxicity by inhibiting the activation of GluN2B-containing NMDA receptors, ultimately resulting in neurotoxic outcomes.

## Electronic Supplementary Material

Below is the link to the electronic supplementary material.

Supplementary Material 1

## Data Availability

No datasets were generated or analysed during the current study.
