# Supplementary material for: Mechanistic Analysis of Decabromodiphenyl Ether-Induced Neurotoxicity in Humans Using Network Toxicology and Molecular Docking
Source: Neurotox Res. 2025 Mar 24;43(2):17. doi: 10.1007/s12640-025-00741-7 (PMC11930881; doi:10.1007/s12640-025-00741-7)
Supplement: Supplementary file 1 — Supplementary Material 1 [file 12640_2025_741_MOESM1_ESM.docx]

**Supplementary Material**

**Mechanistic Analysis of Decabromodiphenyl Ether-Induced Neurotoxicity in Humans Using Network Toxicology and Molecular Docking**

**Supplementary Table Captions**

**Table S1.** Potential 294 targets in BDE-209 induced neurotoxicity in humans.

**Table S2.** Functional data of 294 potential targets ([GeneMANIA](https://genemania.org/search/homo-sapiens/AASS/ABAT/ABCC3/ABCG1/ACBD3/ADAM10/ADNP/AFDN/AFP/AGL/AHCYL2/AHR/AK1/AKT3/ALAS1/ALDH1A1/ALDH5A1/ALDOA/ALPL/APAF1/APOA4/AQP4/AR/ARRB1/ARSA/ATF3/ATF6/ATP7B/BAX/BCHE/BCL2/BDNF/BMP6/BTG2/CALR/CAMK2A/CASP7/CASP9/CAST/CAT/CCL5/CCND1/CD44/CD83/CDC25B/CDK1/CDK5R2/CDKN1A/CEBPA/CEBPB/CHRNA4/CHRNE/CLDN1/CNP/CREB1/CRK/CSNK2A1/CSPG4/CTSL/CTTN/CXCL3/CXCL8/CYP1B1/DAPK1/DBP/DCLK1/DCT/DDR1/DDX18/DIO3/DLAT/DLG1/DLG2/DLG4/DLST/DNAJB1/DNAJC30/DUSP1/EEF2/EEF2K/EGFR/EGR1/EIF4B/EPCAM/EPHB2/EPHX1/EPHX2/FABP3/FAS/FBLN1/FBXO22/FBXO7/FDPS/FECH/FGFR1/FGFR2/FKBP5/FLOT2/FOS/FOSB/FZD5/G6PD/GABBR1/GADD45A/GAP43/GBE1/GCH1/GJA1/GLRA2/GLRA3/GLRX3/GLUL/GNAI1/GOLGA4/GPAM/GPNMB/GPT/GRIA1/GRIA3/GRIK3/GRIN1/GRIN2B/GRM1/GSTM1/GSTZ1/GTF3A/GUCY1A2/HIP1/HK1/HMGA1/HMGB1/HMOX1/HSPA1A/HSPA9/HTR2A/IDH3A/IGF1/IGF2BP2/IGFBP3/IMP4/INA/INPP5A/IRS1/IRS2/ISYNA1/ITSN1/KARS1/KCNA3/KCNMA1/KCNN3/KIF2C/KLC4/KMO/LAMP1/LASP1/LEPR/LGALS3/LIG3/LMNA/LOX/LPL/MACF1/MAP2K2/MAP2K4/MAPK1/MAPRE2/MAPT/MAT2A/MDH2/MT1A/MT2A/MTF2/MYBBP1A/MYLK/NAMPT/NES/NFKB1/NFKBIA/NHERF1/NMNAT1/NQO1/NR1D2/NR4A2/NRG1/NSMAF/NUP98/OCLN/ODC1/PAFAH1B3/PDE1A/PFKM/PGM3/PHLDA1/PILRA/PIP4K2A/PLCB4/PNKP/PRDX5/PRKAA1/PRKCI/PRKD3/PRMT7/PRODH/PSAP/PSIP1/PSMD8/PTPN1/PTX3/PUM3/RAB5A/RELA/RET/RFX5/RGS2/RPS11/RRM1/RTN3/SCG2/SCN7A/SEC23B/SERPINE1/SESN2/SKA2/SLC16A2/SLC22A3/SLC2A10/SLC2A3/SLC44A1/SLC4A3/SLC6A6/SLC8A1/SLCO1C1/SMARCA4/SNAP25/SNCB/SNCG/SOCS1/SOD2/SOD3/SORBS1/SOX2/SP1/SPHK2/SPINK1/SPP1/SPR/SQSTM1/SRD5A1/ST8SIA1/ST8SIA4/STAT3/STC1/STK4/STX1A/STXBP1/SULT1A1/SULT1A4/SYN2/SYT1/TAC1/TFG/TGM2/THBS4/THRA/TIMP3/TJP2/TLR4/TNFRSF21/TNR/TPM4/TRA2A/TRAPPC10/TRH/TRMT61A/TSN/TUBA1B/TUBB2A/TUBB3/TUBB4B/TUBB6/TUBG1/TWF2/UBA3/UBC/UROD/USP13/USP2/VAMP2/VCAM1/VIM/WNT5A/WSB1/ZFP36/ZNF740/)).

**Table S3.** Pathway enrichment analysis of 294 potential targets ([DAVID](https://david.ncifcrf.gov/tools.jsp)).

**Table S4.** Pathway enrichment analysis of four key targets ([DAVID](https://david.ncifcrf.gov/tools.jsp)).

**Supplementary Figure Caption**

**Figure S1.** Gene-gene interaction network of 294 potential targets.

**Table S1.** Potential 294 targets in BDE-209 induced neurotoxicity in humans.

| AASS, ABAT, ABCC3, ABCG1, ACBD3, ADAM10, ADNP, AFDN, AFP, AGL, AHCYL2, AHR, AK1, AKT3, ALAS1, ALDH1A1, ALDH5A1, ALDOA, ALPL, APAF1, APOA4, AQP4, AR, ARRB1, ARSA, ATF3, ATF6, ATP7B, BAX, BCHE, BCL2, BDNF, BMP6, BTG2, CALR, CAMK2A, CASP7, CASP9, CAST, CAT, CCL5, CCND1, CD44, CD83, CDC25B, CDK1, CDK5R2, CDKN1A, CEBPA, CEBPB, CHRNA4, CHRNE, CLDN1, CNP, CREB1, CRK, CSNK2A1, CSPG4, CTSL, CTTN, CXCL3, CXCL8, CYP1B1, DAPK1, DBP, DCLK1, DCT, DDR1, DDX18, DIO3, DLAT, DLG1, DLG2, DLG4, DLST, DNAJB1, DNAJC30, DUSP1, EEF2, EEF2K, EGFR, EGR1, EIF4B, EPCAM, EPHB2, EPHX1, EPHX2, FABP3, FAS, FBLN1, FBXO22, FBXO7, FDPS, FECH, FGFR1, FGFR2, FKBP5, FLOT2, FOS, FOSB, FZD5, G6PD, GABBR1, GADD45A, GAP43, GBE1, GCH1, GJA1, GLRA2, GLRA3, GLRX3, GLUL, GNAI1, GOLGA4, GPAM, GPNMB, GPT, GRIA1, GRIA3, GRIK3, GRIN1, GRIN2B, GRM1, GSTM1, GSTZ1, GTF3A, GUCY1A2, HIP1, HK1, HMGA1, HMGB1, HMOX1, HSPA1A, HSPA9, HTR2A, IDH3A, IGF1, IGF2BP2, IGFBP3, IMP4, INA, INPP5A, IRS1, IRS2, ISYNA1, ITSN1, KARS1, KCNA3, KCNMA1, KCNN3, KIF2C, KLC4, KMO, LAMP1, LASP1, LEPR, LGALS3, LIG3, LMNA, LOX, LPL, MACF1, MAP2K2, MAP2K4, MAPK1, MAPRE2, MAPT, MAT2A, MDH2, MT1A, MT2A, MTF2, MYBBP1A, MYLK, NAMPT, NES, NFKB1, NFKBIA, NHERF1, NMNAT1, NQO1, NR1D2, NR4A2, NRG1, NSMAF, NUP98, OCLN, ODC1, PAFAH1B3, PDE1A, PFKM, PGM3, PHLDA1, PILRA, PIP4K2A, PLCB4, PNKP, PRDX5, PRKAA1, PRKCI, PRKD3, PRMT7, PRODH, PSAP, PSIP1, PSMD8, PTPN1, PTX3, PUM3, RAB5A, RELA, RET, RFX5, RGS2, RPS11, RRM1, RTN3, SCG2, SCN7A, SEC23B, SERPINE1, SESN2, SKA2, SLC16A2, SLC22A3, SLC2A10, SLC2A3, SLC44A1, SLC4A3, SLC6A6, SLC8A1, SLCO1C1, SMARCA4, SNAP25, SNCB, SNCG, SOCS1, SOD2, SOD3, SORBS1, SOX2, SP1, SPHK2, SPINK1, SPP1, SPR, SQSTM1, SRD5A1, ST8SIA1, ST8SIA4, STAT3, STC1, STK4, STX1A, STXBP1, SULT1A1, SULT1A4, SYN2, SYT1, TAC1, TFG, TGM2, THBS4, THRA, TIMP3, TJP2, TLR4, TNFRSF21, TNR, TPM4, TRA2A, TRAPPC10, TRH, TRMT61A, TSN, TUBA1B, TUBB2A, TUBB3, TUBB4B, TUBB6, TUBG1, TWF2, UBA3, UBC, UROD, USP13, USP2, VAMP2, VCAM1, VIM, WNT5A, WSB1, ZFP36, ZNF740 |
| --- |

**Table S2.** Functional data of 294 potential targets ([GeneMANIA](https://genemania.org/search/homo-sapiens/AASS/ABAT/ABCC3/ABCG1/ACBD3/ADAM10/ADNP/AFDN/AFP/AGL/AHCYL2/AHR/AK1/AKT3/ALAS1/ALDH1A1/ALDH5A1/ALDOA/ALPL/APAF1/APOA4/AQP4/AR/ARRB1/ARSA/ATF3/ATF6/ATP7B/BAX/BCHE/BCL2/BDNF/BMP6/BTG2/CALR/CAMK2A/CASP7/CASP9/CAST/CAT/CCL5/CCND1/CD44/CD83/CDC25B/CDK1/CDK5R2/CDKN1A/CEBPA/CEBPB/CHRNA4/CHRNE/CLDN1/CNP/CREB1/CRK/CSNK2A1/CSPG4/CTSL/CTTN/CXCL3/CXCL8/CYP1B1/DAPK1/DBP/DCLK1/DCT/DDR1/DDX18/DIO3/DLAT/DLG1/DLG2/DLG4/DLST/DNAJB1/DNAJC30/DUSP1/EEF2/EEF2K/EGFR/EGR1/EIF4B/EPCAM/EPHB2/EPHX1/EPHX2/FABP3/FAS/FBLN1/FBXO22/FBXO7/FDPS/FECH/FGFR1/FGFR2/FKBP5/FLOT2/FOS/FOSB/FZD5/G6PD/GABBR1/GADD45A/GAP43/GBE1/GCH1/GJA1/GLRA2/GLRA3/GLRX3/GLUL/GNAI1/GOLGA4/GPAM/GPNMB/GPT/GRIA1/GRIA3/GRIK3/GRIN1/GRIN2B/GRM1/GSTM1/GSTZ1/GTF3A/GUCY1A2/HIP1/HK1/HMGA1/HMGB1/HMOX1/HSPA1A/HSPA9/HTR2A/IDH3A/IGF1/IGF2BP2/IGFBP3/IMP4/INA/INPP5A/IRS1/IRS2/ISYNA1/ITSN1/KARS1/KCNA3/KCNMA1/KCNN3/KIF2C/KLC4/KMO/LAMP1/LASP1/LEPR/LGALS3/LIG3/LMNA/LOX/LPL/MACF1/MAP2K2/MAP2K4/MAPK1/MAPRE2/MAPT/MAT2A/MDH2/MT1A/MT2A/MTF2/MYBBP1A/MYLK/NAMPT/NES/NFKB1/NFKBIA/NHERF1/NMNAT1/NQO1/NR1D2/NR4A2/NRG1/NSMAF/NUP98/OCLN/ODC1/PAFAH1B3/PDE1A/PFKM/PGM3/PHLDA1/PILRA/PIP4K2A/PLCB4/PNKP/PRDX5/PRKAA1/PRKCI/PRKD3/PRMT7/PRODH/PSAP/PSIP1/PSMD8/PTPN1/PTX3/PUM3/RAB5A/RELA/RET/RFX5/RGS2/RPS11/RRM1/RTN3/SCG2/SCN7A/SEC23B/SERPINE1/SESN2/SKA2/SLC16A2/SLC22A3/SLC2A10/SLC2A3/SLC44A1/SLC4A3/SLC6A6/SLC8A1/SLCO1C1/SMARCA4/SNAP25/SNCB/SNCG/SOCS1/SOD2/SOD3/SORBS1/SOX2/SP1/SPHK2/SPINK1/SPP1/SPR/SQSTM1/SRD5A1/ST8SIA1/ST8SIA4/STAT3/STC1/STK4/STX1A/STXBP1/SULT1A1/SULT1A4/SYN2/SYT1/TAC1/TFG/TGM2/THBS4/THRA/TIMP3/TJP2/TLR4/TNFRSF21/TNR/TPM4/TRA2A/TRAPPC10/TRH/TRMT61A/TSN/TUBA1B/TUBB2A/TUBB3/TUBB4B/TUBB6/TUBG1/TWF2/UBA3/UBC/UROD/USP13/USP2/VAMP2/VCAM1/VIM/WNT5A/WSB1/ZFP36/ZNF740/)).

| **Function** | **FDR value** | **Gene in network** | **Gene in genome** |
| --- | --- | --- | --- |
| cellular response to chemical stress | 1.50x10^-11^ | 28 | 253 |
| response to oxidative stress | 3.78x10^-11^ | 27 | 248 |
| cellular response to oxidative stress | 4.35x10^-11^ | 22 | 156 |
| regulation of apoptotic signaling pathway | 1.62x10^-08^ | 24 | 255 |
| neuron death | 2.84x10^-08^ | 21 | 197 |
| extrinsic apoptotic signaling pathway | 6.71x10^-08^ | 18 | 146 |
| regulation of extrinsic apoptotic signaling pathway | 6.94x10^-08^ | 16 | 111 |
| response to reactive oxygen species | 1.57x10^-07^ | 16 | 118 |
| regulation of MAP kinase activity | 2.20x10^-07^ | 22 | 250 |
| epithelial cell proliferation | 6.55x10^-07^ | 22 | 266 |

**Table S3.** Pathway enrichment analysis of 294 potential targets ([DAVID](https://david.ncifcrf.gov/tools.jsp)).

| **REACTOME term** | ***p* value** | **Gene count** |
| --- | --- | --- |
| Transmission across Chemical Synapses | 2.20x10^-15^ | 35 |
| Neuronal System | 4.00x10^-14^ | 41 |
| Post NMDA receptor activation events | 6.70x10^-11^ | 17 |
| Activation of NMDA receptors and postsynaptic events | 7.60x10^-11^ | 18 |
| Neurotransmitter receptors and postsynaptic signal transmission | 1.70x10^-10^ | 25 |
| Cellular responses to stimuli | 8.50x10^-09^ | 52 |
| Cytokine Signaling in Immune system | 1.30x10^-08^ | 47 |
| KEAP1-NFE2L2 pathway | 1.30x10^-08^ | 17 |
| Cellular responses to stress | 3.10x10^-08^ | 47 |
| Assembly and cell surface presentation of NMDA receptors | 3.60x10^-08^ | 11 |

**Table S4.** Pathway enrichment analysis of four key targets ([DAVID](https://david.ncifcrf.gov/tools.jsp)).

| **REACTOME term** | ***p* value** | **Genes** | **Count** |
| --- | --- | --- | --- |
| Unblocking of NMDA receptors, glutamate binding and activation | 6.70x10^-09^ | CAMK2A/DLG4/GRIA1/GRIN2B | 4 |
| Long-term potentiation | 8.80x10^-09^ | CAMK2A/DLG4/GRIA1/GRIN2B | 4 |
| Post NMDA receptor activation events | 3.60x10^-07^ | CAMK2A/DLG4/GRIA1/GRIN2B | 4 |
| Activation of NMDA receptors and postsynaptic events | 5.60x10^-07^ | CAMK2A/DLG4/GRIA1/GRIN2B | 4 |
| Neurotransmitter receptors and postsynaptic signal transmission | 6.10x10^-06^ | CAMK2A/DLG4/GRIA1/GRIN2B | 4 |
| Ras activation upon Ca2+ influx through NMDA receptor | 1.00x10^-05^ | CAMK2A/DLG4/GRIN2B | 3 |
| Synaptic adhesion-like molecules | 1.00x10^-05^ | DLG4/GRIA1/GRIN2B | 3 |
| Negative regulation of NMDA receptor-mediated neuronal transmission | 1.10x10^-05^ | CAMK2A/DLG4/GRIN2B | 3 |
| Transmission across Chemical Synapses | 1.40x10^-05^ | CAMK2A/DLG4/GRIA1/GRIN2B | 4 |
| CREB1 phosphorylation through NMDA receptor-mediated activation of RAS signaling | 2.00x10^-05^ | CAMK2A/DLG4/GRIN2B | 3 |

**
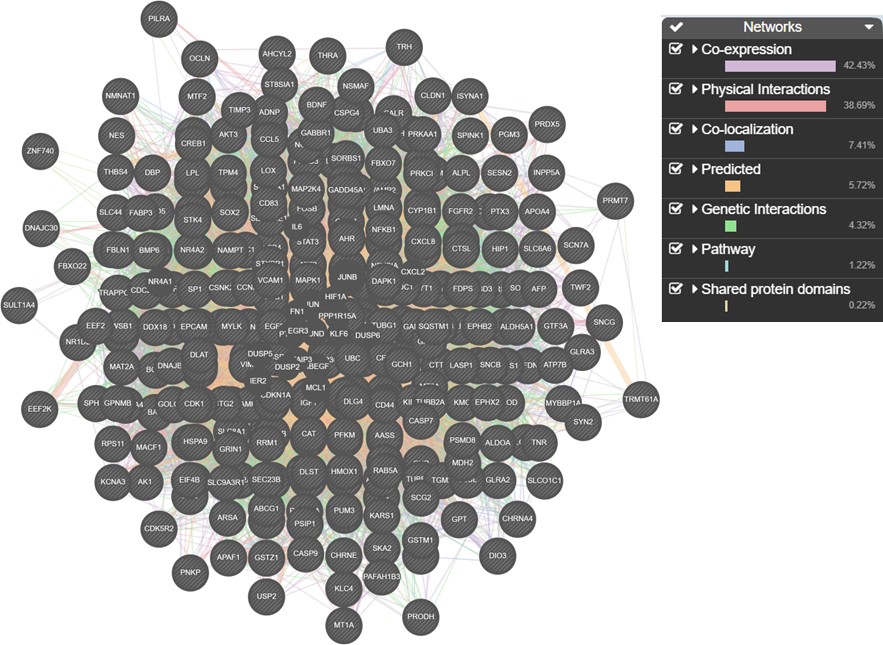
**

**Figure S1.** Gene-gene interaction network of 294 potential targets.
